# Molecular and Clinical Characterization of LIGHT/TNFSF14 Expression at Transcriptional Level *via* 998 Samples With Brain Glioma

**DOI:** 10.3389/fmolb.2021.567327

**Published:** 2021-08-27

**Authors:** Ying Yang, Wen Lv, Shihai Xu, Fei Shi, Aijun Shan, Jin Wang

**Affiliations:** ^1^ Department of Pediatrics, Futian Women and Children Institute, Shenzhen, China; ^2^ Emergency Department, Shenzhen People’s Hospital (The Second Clinical Medical College of Jinan University, The First Affiliated Hospital of Southern University of Science and Technology), Shenzhen, China

**Keywords:** glioma, LIGHT, TNFSF14, immune response, prognosis

## Abstract

LIGHT, also termed TNFSF14, has been reported to play a vital role in different tumors. However, its role in glioma remains unknown. This study is aimed at unveiling the characterization of the transcriptional expression profiling of LIGHT in glioma. We selected 301 glioma patients with mRNA microarray data from the CGGA dataset and 697 glioma patients with RNAseq data from the TCGA dataset. Transcriptome data and clinical data of 998 samples were analyzed. Statistical analyses and figure generation were performed with R language. LIGHT expression showed a positive correlation with WHO grade of glioma. LIGHT was significantly increased in mesenchymal molecular subtype. Gene Ontology analysis demonstrated that LIGHT was profoundly involved in immune response. Moreover, LIGHT was found to be synergistic with various immune checkpoint members, especially HVEM, PD1/PD-L1 pathway, TIM3, and B7-H3. To get further understanding of LIGHT-related immune response, we put LIGHT together with seven immune signatures into GSVA and found that LIGHT was particularly correlated with HCK, LCK, and MHC-II in both datasets, suggesting a robust correlation between LIGHT and activities of macrophages, T-cells, and antigen-presenting cells (APCs). Finally, higher LIGHT indicated significantly shorter survival for glioma patients. Cox regression models revealed that LIGHT expression was an independent variable for predicting survival. In conclusion, LIGHT was upregulated in more malignant gliomas including glioblastoma, IDH wildtype, and mesenchymal subtype. LIGHT was mainly involved in the immune function of macrophages, T cells, and APCs and served as an independent prognosticator in glioma.

## Introduction

In the brain, glioma accounts for the most common and malignant primary tumor in adult patients (Meng et al., 2020). Despite improvements in diagnosis and treatment, the prognosis of glioma remains unfavorable, especially in glioblastoma (GBM), the most aggressive and malignant type (Wang et al., 2019a; Wei et al., 2019). Recent advancements in immunotherapy have brought a new hope for tumor treatment. Identifying novel immune targets may facilitate the development of new immunotherapeutic strategies in glioma.

LIGHT, also termed tumor necrosis factor superfamily member 14 (TNFSF14), has been widely reported in a range of malignancies, including non-small-cell lung cancer (NSCLC) (Brunetti et al., 2020), multiple myeloma (Brunetti et al., 2014; Brunetti et al., 2018), colorectal cancer (Qin et al., 2013; Maker et al., 2015; Qiao et al., 2017), prostate tumor (Yan et al., 2015), breast cancer (Gantsev et al., 2013), and melanoma (Hehlgans and Männel, 2001). LIGHT has been reported to play a dualistic role (anti-tumor or pro-tumor) in different tumors. In melanoma (Hehlgans and Männel, 2001), colorectal cancer (Maker et al., 2015; Qiao et al., 2017), and prostate cancer (Yan et al., 2015), a higher level of LIGHT expression was associated with a better prognosis. While for patients with NSCLC (Brunetti et al., 2020), multiple myeloma (Brunetti et al., 2014; Brunetti et al., 2018), and breast cancer (Gantsev et al., 2013), an increased pattern of LIGHT expression predicted a much worse survival.

To date, the authors failed to find a comprehensive study about the characterization of LIGHT expression in pan-glioma. Only one article reported by Long et al. (2020) described the role and prognostic value of LIGHT in GBM. In the present study, transcriptome data of 998 pan-glioma patients were analyzed to demonstrate the expression profile of LIGHT in pan-glioma molecularly and clinically.

## Materials and Methods

### Sample and Data Collection

Transcriptome data and clinical data of glioma patients were available on the Chinese Glioma Genome Atlas (CGGA) website (http://www.cgga.org.cn/) and TCGA website (http://cancergenome.nih.gov/). A total of 998 glioma patients, including 301 CGGA microarray data (GeneSpring GX 11.0 normalization) and 697 TCGA RNA sequencing data (RSEM normalization, level 3), were enrolled. The baseline characteristics of patients in both cohorts are described in Supplementary Table S1. This study was approved by the Ethics Committee of Shenzhen People’s Hospital.

### Statistical Analysis

Before analysis, transcriptome data were pre-processed. For TCGA data, RSEM-normalized RNA sequencing data were log2 transformed. For CGGA data, microarray data (already normalized and centered by a data provider) were directly enrolled and analyzed. Statistical analysis was performed with R language. Multiple R packages, including ggplot2, pROC, pheatmap, corrgram, circlize, and survival, were used to generate figures. The biological processes of LIGHT-related genes were annotated using Gene Ontology (GO) (DAVID, https://david.ncifcrf.gov/) enrichment analysis. The Cox proportional hazard regression analyses were performed with coxph function available in the survival package. All statistical tests were two-sided. A *p* value less than 0.05 was taken as significant.

## Results

### LIGHT Expression Was Positively Correlated With WHO Grade of Glioma and Increased in IDH Wildtype Glioma

**FIGURE 1:**
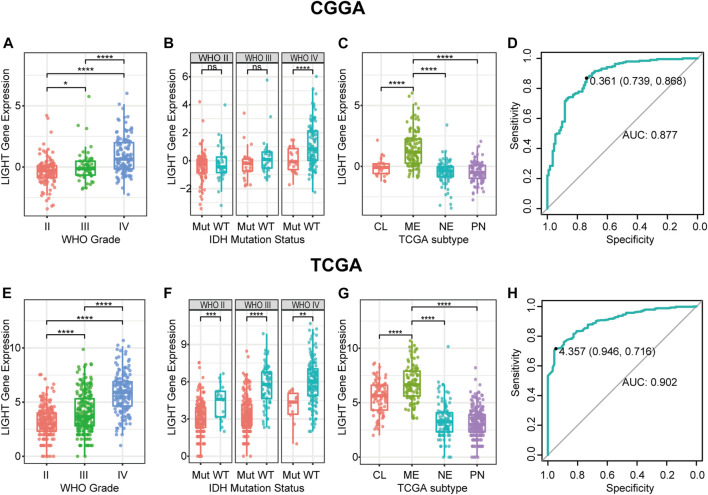
LIGHT expression in CGGA and TCGA datasets according to WHO grade **(A**, **E)**, IDH mutation status **(B**, **F)**, TCGA molecular subtype **(C**, **G)**, and ROC curves **(D**, **H)** for distinguishing mesenchymal subtypes. * indicates *p* value <0.05, **indicates *p* value <0.01, *** indicates *p* value <0.001, and **** indicates *p* value <0.0001.

LIGHT expression levels were compared across different WHO grades. The results of both CGGA and TCGA cohorts consistently showed a statistically significant positive correlation between LIGHT expression and WHO grade of glioma (Figures 1A, E), suggesting that a higher LIGHT level was paralleled with higher malignancy in glioma. In addition, when patients were further subclassified with respect to IDH mutation status, IDH wildtype glioma was found to be more associated with an increased pattern of LIGHT expression in both datasets, though a statistical significance was not detected in some subgroups (Figures 1B, F). This further indicated that LIGHT was particularly associated with a more aggressive biological behavior in glioma.

### LIGHT Was Upregulated in Mesenchymal Subtype

To examine the relationship between LIGHT expression and molecular subtype, we analyzed LIGHT expression according to the molecular classification scheme of glioma defined by the TCGA project. As shown in Figures 1C, G, LIGHT expression in mesenchymal molecular subtype was significantly upregulated than that in other subtypes. The results suggested that LIGHT expression could serve as a specific biomarker for the mesenchymal subtype of glioma. ROC curves were further performed to evaluate the performance of LIGHT expression for distinguishing mesenchymal subtypes. The areas under the ROC curves (AUCs) were 87.7% in the CGGA and 90.2% in TCGA, respectively (Figures 1D, H).

### LIGHT-Related Biological Process

**FIGURE 2:**
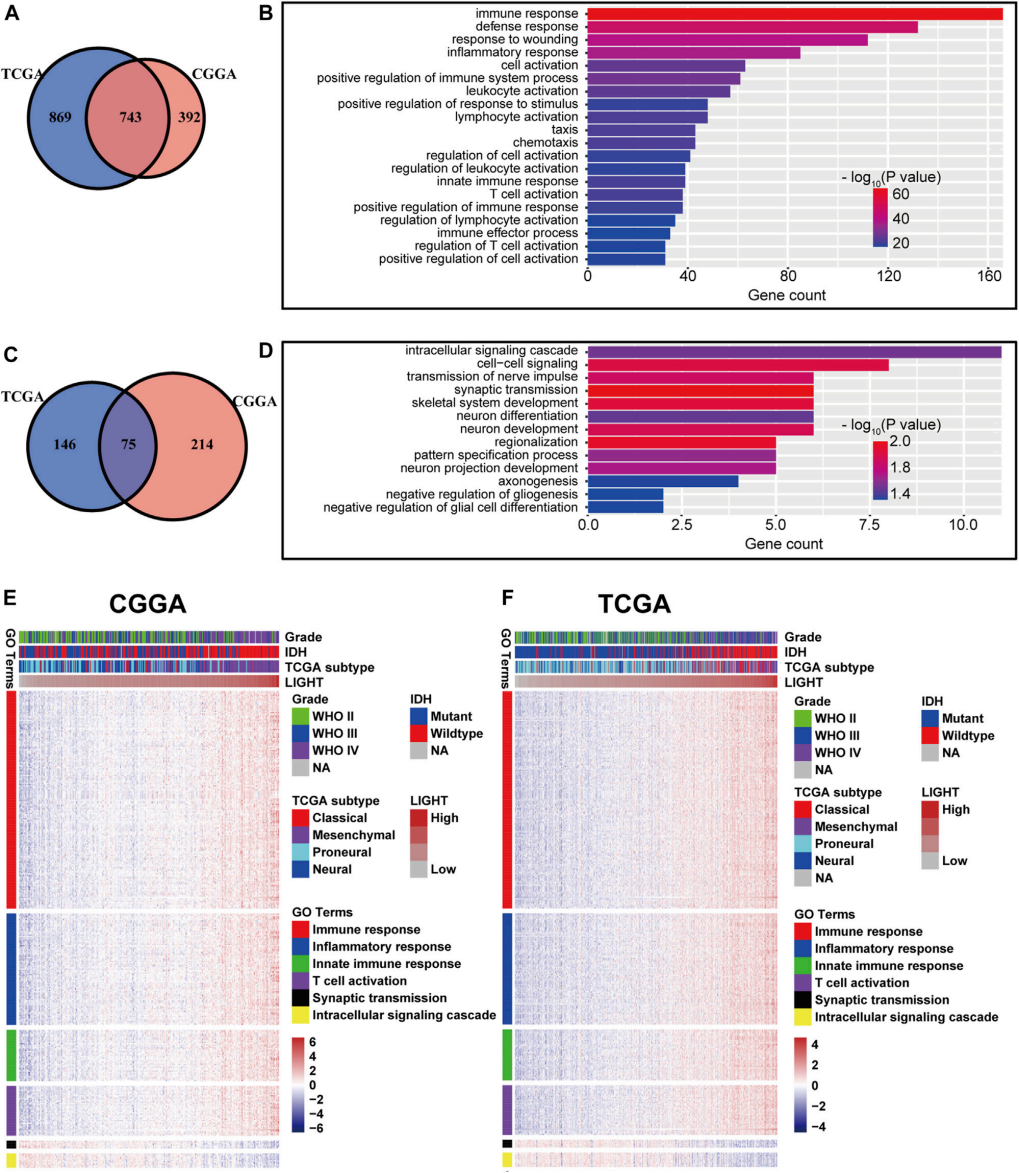
Gene Ontology analysis for LIGHT in pan-glioma. Number of LIGHT positively correlated genes **(A)** and corresponding biological processes **(B);** number of LIGHT negatively correlated genes **(C)** and corresponding biological processes **(D)**; clusters of GO terms of LIGHT highly related genes in the CGGA **(E)** and TCGA **(F)**.

To explore the biological features of LIGHT in glioma, the Pearson correlation test was performed between LIGHT and every single gene. With the criteria of Pearson coefficient |r| > 0.5, we identified 1135 LIGHT positively correlated genes and 289 LIGHT negatively correlated genes in the CGGA and 1612 LIGHT positively correlated genes and 221 LIGHT negatively correlated genes in TCGA (Figures 2A, C). To ensure the accuracy of results, the overlapped LIGHT-related genes in both CGGA and TCGA cohorts were selected. Gene Ontology (GO) (DAVID 6.8 version, https://david.ncifcrf.gov/) analysis was utilized to determine the biological process of LIGHT-related genes. The results showed that LIGHT positively correlated genes were mainly associated with immune response and inflammatory response, particularly involvement in T-cell activation (Figures 2B, E, and F). In contrast, LIGHT negatively correlated genes were more associated with normal biological function, such as synaptic transmission and intracellular signaling cascade (Figures 2D–F). Moreover, GO analysis in GBM revealed that LIGHT-related biological process showed a similar pattern to that in pan-glioma (Supplementary Figure S1). These results indicated that LIGHT was induced as an immune suppressor in glioma in which tumor-related immune and inflammatory responses were activated.

### LIGHT Interacted With Immune Checkpoint Members in Glioma

**FIGURE 3:**
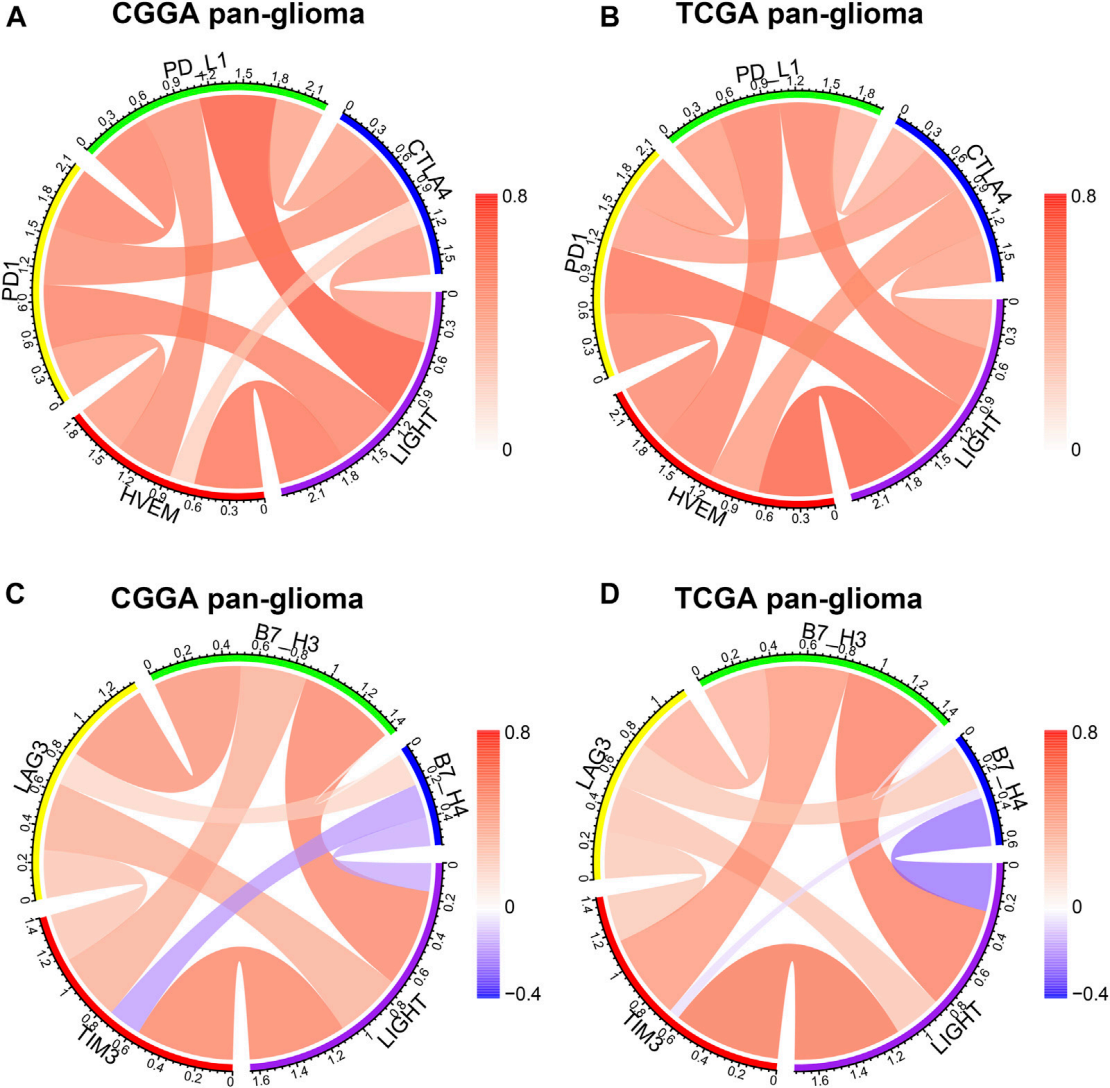
Correlation of LIGHT and immune checkpoint members in pan-glioma. Correlation of LIGHT and HVEM, PD1, PD-L1, CTLA4 **(A, B)**. Correlation of LIGHT and TIM3, LAG3, B7-H3, B7-H4 **(C, D)**.

LIGHT is one of the ligands of herpes virus entry mediator (HVEM, also termed TNFRSF14), and the conjugation of two molecules facilitates anti-tumoral or pro-tumoral immunity, depending on the histological type of cancer (Han et al., 2019). As annotated in the GO analysis, LIGHT plays an immunosuppressive role in glioma. To further validate the pro-tumoral role of LIGHT in glioma, we performed the Pearson correlation test to examine the relationship between LIGHT and immune checkpoint members, including HVEM, CTLA-4, PD-L1, PD-1, TIM3, LAG3, B7-H3, and B3-H4. In whole grade glioma of the CGGA and TCGA, Circos plots based on the Pearson r value revealed that LIGHT expression was significantly associated with these immune checkpoint members, especially HVEM, PD1/PD-L1 pathway, TIM-3, and B7-H3 (Figures 3A–D). To further demonstrate the intercorrelation of these immune checkpoint members in GBM, Pearson correlation tests were additionally performed. As shown in Supplementary Figures S2A–D, in line with pan-glioma, the correlation between LIGHT and these immune checkpoints in GBM was also very robust in both datasets, indicating synergistic effects of these markers during glioma-related immune response.

### LIGHT-Related Immune and Inflammatory Activities

**FIGURE 4:**
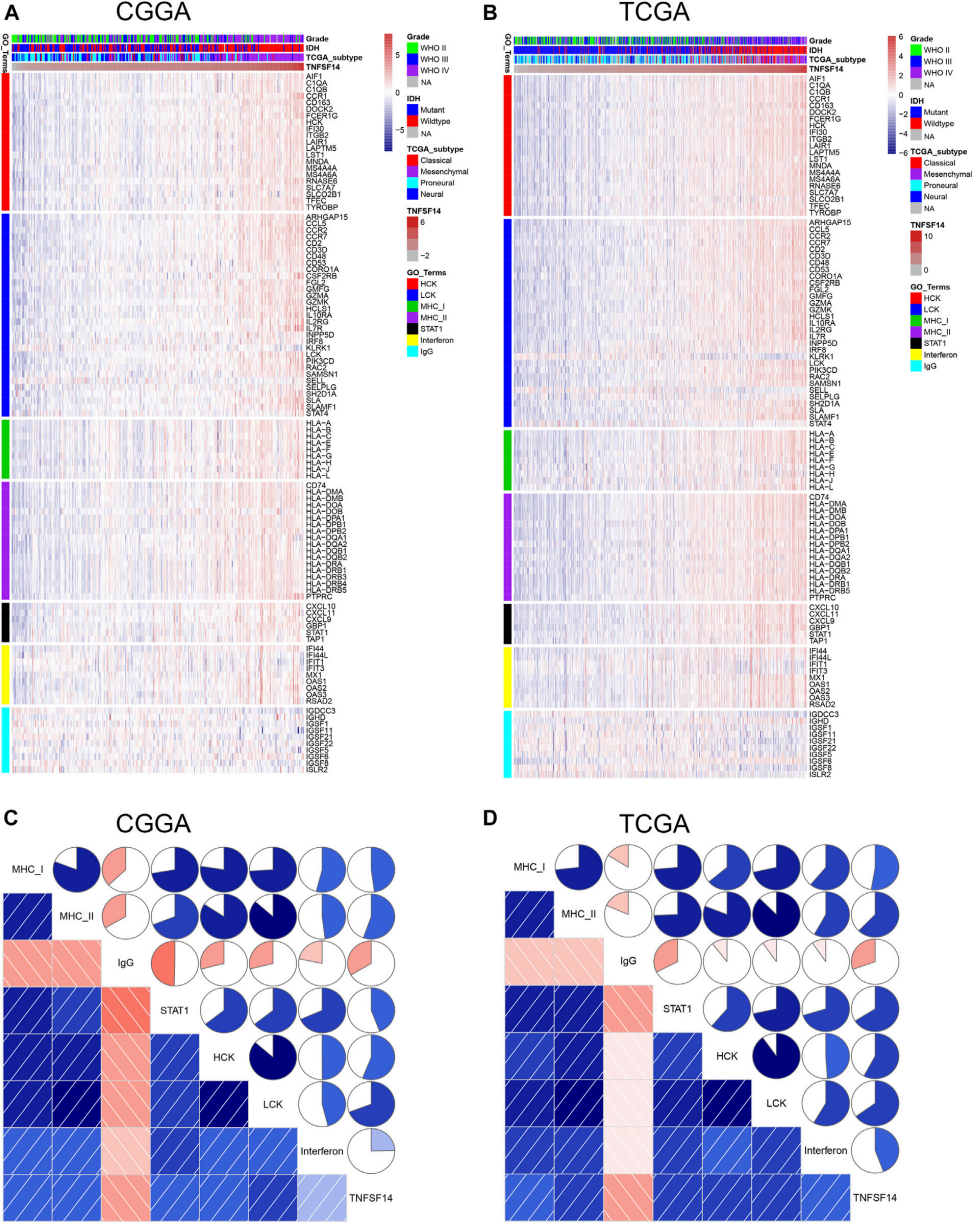
Clusters **(A**, **B)** and gene set variation analysis **(C**, **D)** of LIGHT-related immune response.

To further investigate LIGHT-related immune and inflammatory activities, seven clusters, including 104 genes representing different types of immune and inflammatory activities, were defined as metagenes (Rody et al., 2009) (Supplementary Table S2) and subsequently put into gene set variation analysis (GSVA) (Hänzelmann et al., 2013). As indicated in Figures 4A, B, LIGHT expression was found to be positively associated with the majority of clusters, except for IgG, which was specific for B-cell immune activity. Corrgram plots derived from GSVA results demonstrated that LIGHT was positively correlated with MHC-I, MHC-II, HCK, LCK, STAT1, and interferon, especially HCK, LCK, and MHC-II (Figures 4C, D), which specifically reflected activities of macrophages, T-cells, and antigen-presenting cells (APCs), respectively. These results suggested that LIGHT might be an immunosuppressive molecule in the activities of macrophages, T-cells, and APCs in glioma.

### Higher LIGHT Predicted Shorter Survival for Glioma

**FIGURE 5:**
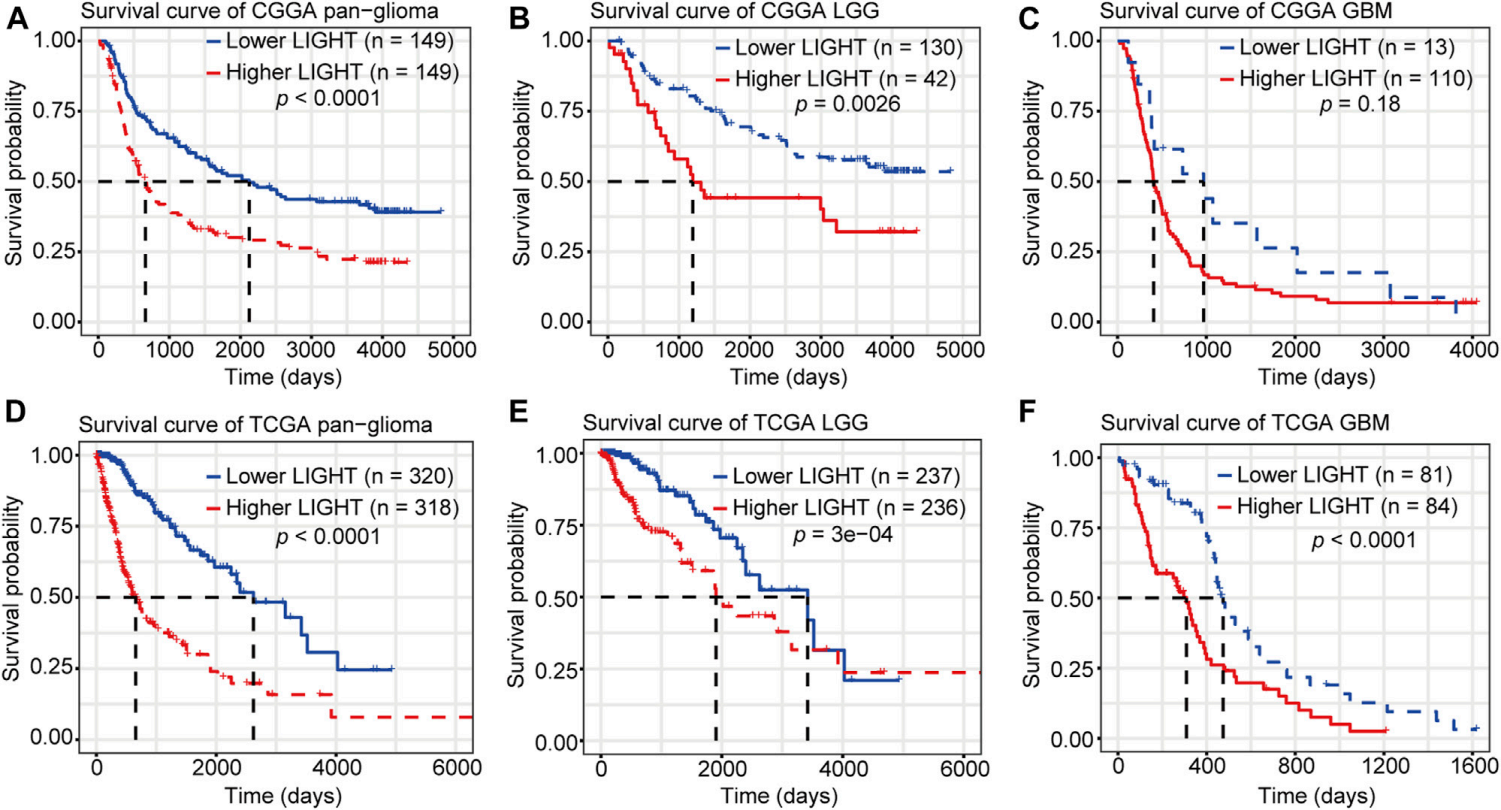
Survival analysis for LIGHT in pan-glioma **(A**, **D)**, LGG **(B**, **E)**, and GBM **(C**, **F)**.

Kaplan–Meier (KM) survival analyses were performed to examine the prognostic role of LIGHT in glioma. According to LIGHT expression, whole grade glioma patients were divided into two groups in each dataset. As shown in Figures 5A, D, higher level of LIGHT expression predicted a significantly shorter survival among pan-glioma patients. Moreover, a similar pattern of the KM survival curve was observed among patients with LGG (Figures 5B, E) and GBM (Figures 5C, F), though no statistical significance was detected in GBM of the CGGA cohort (*p* = 0.18), which also showed an apparent trend (Figure 5C). To further investigate the prognostic value of LIGHT, we performed a multivariate Cox proportional hazard regression analysis using variables including age, WHO grade, IDH mutation status, and LIGHT expression. In the TCGA dataset, LIGHT expression was independently associated with poor prognosis in glioma (TCGA: *p* = 0.005, HR = 1.148), suggesting that LIGHT may serve as a significant prognosticator for glioma patients. In contrast, in the CGGA dataset, no independent association between LIGHT and prognosis was observed (CGGA: *p* = 0.324, HR = 1.170), which may be accounted for collinearity between LIGHT and WHO grade (Tables 1, 2).

**TABLE 1:** Cox logistic regression analysis of overall survival in the CGGA_301 cohort.

Covariates	Univariate			Multivariate		
	HR	95% CI	*p* value	HR	95% CI	*p* value
Age (years)	1.041	1.027–1.055	0.000	1.018	1.005–1.032	0.007
WHO grade (II–IV)	2.670	2.221–3.210	0.000	2.326	1.878–2.880	0.000
IDH (ref. mutant type)	2.541	1.869–3.454	0.000	1.176	0.825–1.677	0.371
LIGHT (ref. lower LIGHT)	1.795	1.342–2.401	0.000	1.170	0.857–1.597	0.324

**TABLE 2:** Cox logistic regression analysis of overall survival in the TCGA cohort.

Covariates	Univariate			Multivariate		
	HR	95% CI	*p* value	HR	95% CI	*p* value
Age (years)	1.075	1.062–1.087	0.000	1.044	1.028–1.059	0.000
WHO grade (II–IV)	5.057	3.915–6.532	0.000	1.961	1.434–2.682	0.000
IDH (ref. mutant type)	13.101	8.962–19.150	0.000	3.620	2.136–6.135	0.000
LIGHT (ref. lower LIGHT)	1.460	1.340–1.590	0.000	1.148	1.043–1.262	0.005

## Discussion

Immunotherapy has brought new hopes for patients with glioma and other malignancies (Li et al., 2017; Wang et al., 2019b). Identifying novel molecular targets for immunotherapy would help not only to distinguish patients who are more likely to benefit but also to overcome resistance and improve efficacy. LIGHT, as a member of the immune costimulatory family, arouses interest for its dualistic behavior in oncogenesis across different tumors. However, in glioma, the role of LIGHT remains unknown.

When we finished this manuscript, we found a study about LIGHT expression in GBM, which was presented by Long et al. (2020). To further unravel the characterization and prognostic value of LIGHT in whole grades of glioma, we performed this analysis as a comprehensive study. In the present study, we investigated the transcriptional expression profiles of LIGHT in 998 glioma patients and revealed that LIGHT expression showed a significantly positive correlation with WHO grade of glioma. Furthermore, higher LIGHT expression in the tumor microenvironment (TME) was usually accompanied by a more aggressive and malignant phenotype in glioma, including GBM, IDH wildtype, and mesenchymal subtype. Moreover, higher LIGHT expression indicated a significantly shorter survival for patients with glioma across different WHO grades. These findings suggested that LIGHT played a pro-tumoral role in glioma, in line with the previous study presented by Long et al. (2020). Understanding the molecular mechanism of LIGHT in glioma may provide novel therapeutic targets to overcome this fatal disease.

Through GO analysis and GSVA, LIGHT was found to play a suppressive role in immune response, especially in activities of macrophages, T cells, and APCs. The immunosuppressive effect of LIGHT may be related to conjugation with its receptor—HVEM. Our Circos plots revealed that LIGHT showed a robust correlation with HVEM, indicating an active LIGHT/HVEM pathway in glioma. The HVEM gene was also reported as an oncogene in glioma (Han et al., 2019). However, the mechanism of LIGHT/HVEM signaling pathway in pro-gliomagenesis remains unclear. As a canonical TNF/TNFR pathway, the LIGHT/HVEM pathway activates nuclear factor-κB (NF-κB) transcription factors through TNF receptor–associated factors (TRAFs), which subsequently controls transcription of genes essential for cell survival and inflammation (Cheung et al., 2009; Chen and Flies, 2013; Ware and Šedý, 2011; D'Ignazio et al., 2018). In addition, our results revealed that LIGHT played a synergistic role in glioma-induced immune response together with the PD1/PD-L1 pathway, TIM3, and B7-H3, as well as other immune checkpoint members. These results were in line with those of Long et al. (2020) and Wu et al. (2019). They concluded that LIGHT was one of the most important immune checkpoints in GBM. Thus, theoretically, therapies targeting the LIGHT pathway might have synergistic effects as immune checkpoint inhibitors, warranting further experimental validation in the future.

About the work presented by Long et al. (2020) in aging, they have performed an impressive study, focusing on the characterization of LIGHT in GBM. The present study extended the analysis to whole WHO grades, which further elucidated the vital role of LIGHT in glioma. However, a limitation of the current study was that no experimental validation was performed. Further *in vitro* and *in vivo* studies are needed to validate its immunosuppressive role in glioma.

## Data Availability

The datasets presented in this study can be found in online repositories. The names of the repository/repositories and accession number(s) can be found in the article/Supplementary Material.
