# Supplementary material for: Molecular and Clinical Characterization of LIGHT/TNFSF14 Expression at Transcriptional Level via 998 Samples With Brain Glioma
Source: Front Mol Biosci. 2021 Aug 27;8:567327. doi: 10.3389/fmolb.2021.567327 (PMC8430338; doi:10.3389/fmolb.2021.567327)
Supplement: Supplementary file 1 [file DataSheet1.docx]

Supplementary Material

# Supplementary Figures
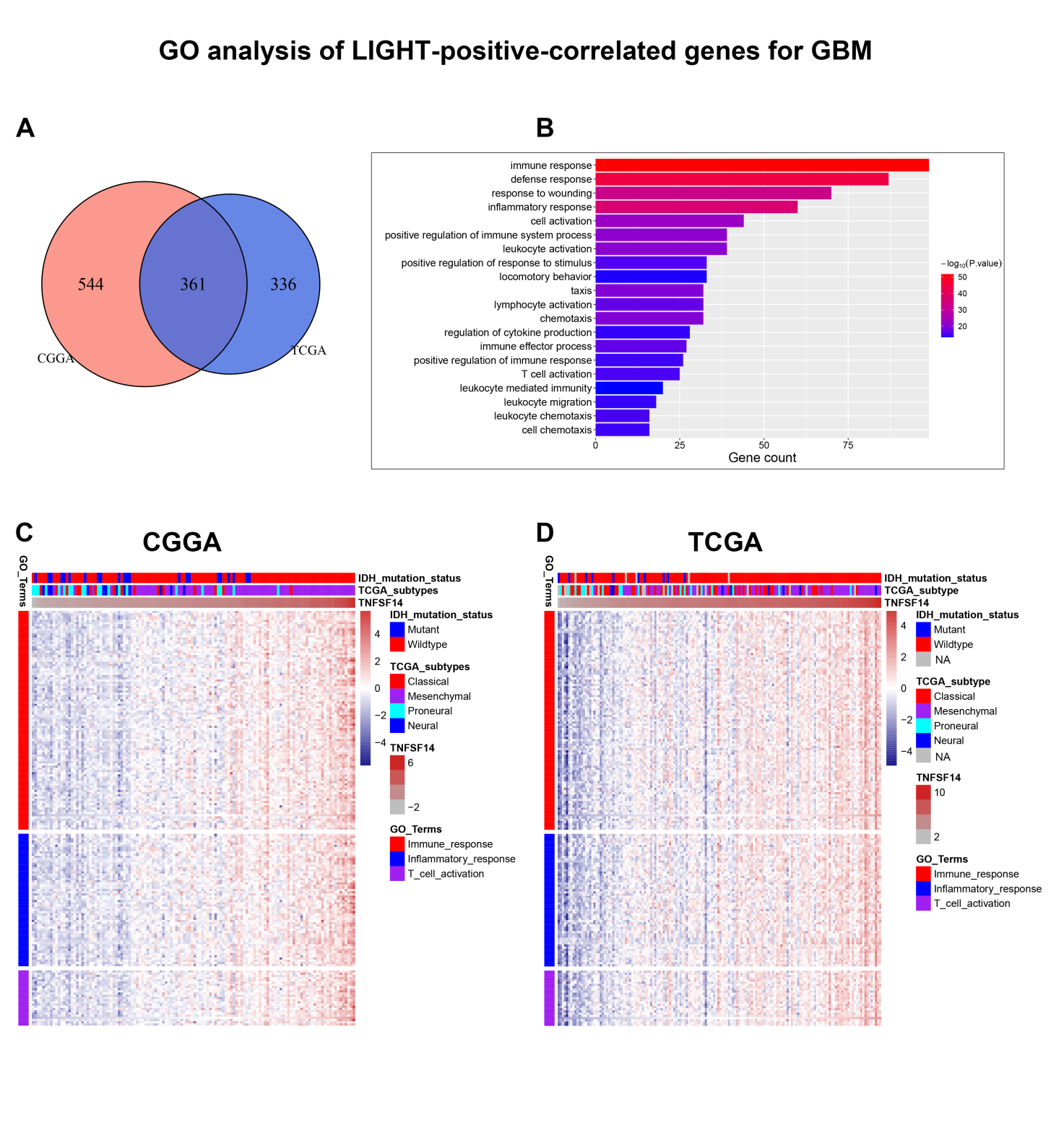


**Supplementary Figure 1.** Gene Ontology analysis for LIGHT in glioblastoma (GBM). Number of LIGHT-positively-correlated genes (A) and corresponding biological processes (B); Clusters of GO terms of LIGHT-highly-related genes in CGGA (C) and TCGA (D).


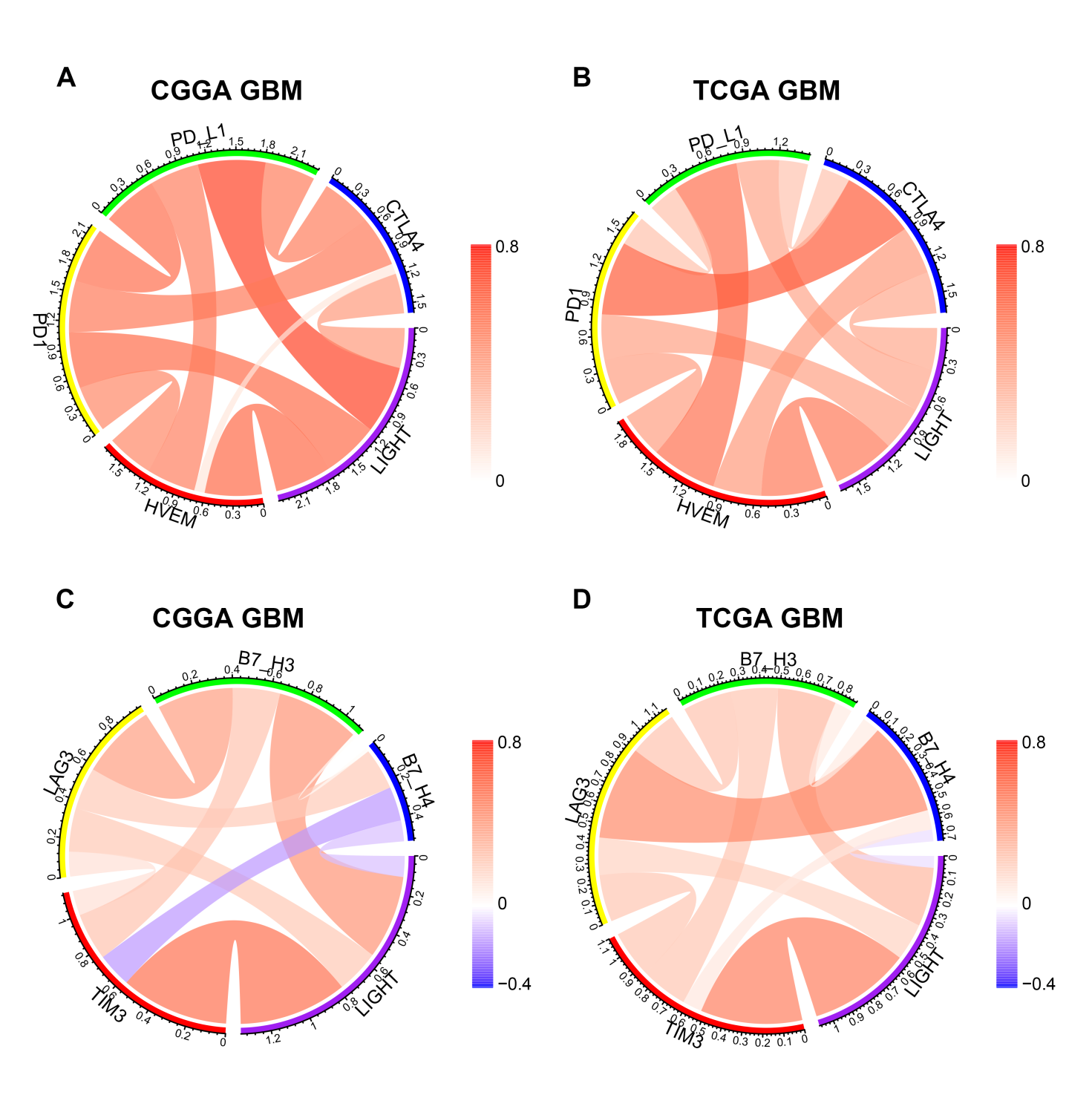


**Supplementary Figure 2.** Correlation of LIGHT and immune checkpoint members in GBM.

# Supplementary Tables

**Supplementary Table 1. Patient characteristics in the TCGA RNA-seq and CGGA_301 microarray data.**

| **Characteristics** | **TCGA RNA-seq (n=697)** | **CGGA microarray (n=301)** |
| --- | --- | --- |
| **Gender** |  |  |
| male | 370 | 180 |
| female | 271 | 121 |
| NA | 56 | 0 |
| **Age (year)** | 47 ± 15 | 42 ± 12 |
| **Tumor subtype** |  |  |
| Classical | 90 | 23 |
| Mesenchymal | 104 | 111 |
| Proneural | 248 | 86 |
| Neural | 115 | 81 |
| NA | 140 | 0 |
| **WHO grade** |  |  |
| Grade II | 226 | 122 |
| Grade III | 249 | 51 |
| Grade IV | 167 | 128 |
| NA | 55 | 0 |
| **Karnofsky Performance Score** | 84 ± 14 | NA |
| **IDH mutation status** |  |  |
| Mut | 442 | 134 |
| WT | 245 | 165 |
| NA | 10 | 2 |
| **1p/19q Codeletion status** |  |  |
| Codeletion | 181 | 16 |
| Non-codeletion | 491 | 76 |
| NA | 25 | 209 |
| **MGMT promoter status** |  |  |
| Methylated | 461 | 99 |
| Unmethylated | 162 | 187 |
| NA | 74 | 15 |

NA: Not Available; KPS: Karnofsky Performance Score; MGMT: O^6^-Methylguanine Methyltransferase

**Supplementary Table 2. Metagenes in the Gene Sets Variation Analysis**

| **Metagenes** | **Genes** |
| --- | --- |
| HCK | C1QB |
| HCK | C1QA |
| HCK | AIF1 |
| HCK | LST1 |
| HCK | DOCK2 |
| HCK | LAPTM5 |
| HCK | TYROBP |
| HCK | MS4A4A |
| HCK | MS4A6A |
| HCK | CD163 |
| HCK | ITGB2 |
| HCK | SLC7A7 |
| HCK | LAIR1 |
| HCK | HCK |
| HCK | TFEC |
| HCK | IFI30 |
| HCK | MNDA |
| HCK | FCER1G |
| HCK | RNASE6 |
| HCK | SLCO2B1 |
| HCK | CCR1 |
| IgG | IGSF8 |
| IgG | ISLR2 |
| IgG | IGSF21 |
| IgG | IGSF1 |
| IgG | IGSF22 |
| IgG | IGDCC3 |
| IgG | IGHD |
| IgG | IGSF11 |
| IgG | IGSF5 |
| IgG | IGSF6 |
| Interferon | IFIT1 |
| Interferon | IFIT3 |
| Interferon | IFI44L |
| Interferon | OAS3 |
| Interferon | MX1 |
| Interferon | RSAD2 |
| Interferon | IFI44 |
| Interferon | OAS2 |
| Interferon | OAS1 |
| LCK | CD2 |
| LCK | GZMK |
| LCK | GZMA |
| LCK | CD3D |
| LCK | CD53 |
| LCK | LCK |
| LCK | ARHGAP15 |
| LCK | CCL5 |
| LCK | GMFG |
| LCK | SELL |
| LCK | STAT4 |
| LCK | SAMSN1 |
| LCK | RAC2 |
| LCK | HCLS1 |
| LCK | CCR7 |
| LCK | PIK3CD |
| LCK | CORO1A |
| LCK | CD48 |
| LCK | IL2RG |
| LCK | SH2D1A |
| LCK | SLAMF1 |
| LCK | IL7R |
| LCK | INPP5D |
| LCK | KLRK1 |
| LCK | FGL2 |
| LCK | IRF8 |
| LCK | SELPLG |
| LCK | IL10RA |
| LCK | SLA |
| LCK | CCR2 |
| LCK | CSF2RB |
| MHC_I | HLA-E |
| MHC_I | HLA-H |
| MHC_I | HLA-B |
| MHC_I | HLA-J |
| MHC_I | HLA-F |
| MHC_I | HLA-G |
| MHC_I | HLA-A |
| MHC_I | HLA-C |
| MHC_I | HLA-L |
| MHC_II | HLA-DRB1 |
| MHC_II | HLA-DRB5 |
| MHC_II | HLA-DRB3 |
| MHC_II | HLA-DPA1 |
| MHC_II | HLA-DRA |
| MHC_II | HLA-DQA1 |
| MHC_II | HLA-DQA2 |
| MHC_II | HLA-DMA |
| MHC_II | HLA-DOA |
| MHC_II | HLA-DRB4 |
| MHC_II | HLA-DMB |
| MHC_II | HLA-DQB1 |
| MHC_II | HLA-DPB1 |
| MHC_II | HLA-DQB2 |
| MHC_II | CD74 |
| MHC_II | PTPRC |
| MHC_II | HLA-DOB |
| MHC_II | HLA-DPB2 |
| STAT1 | TAP1 |
| STAT1 | STAT1 |
| STAT1 | CXCL10 |
| STAT1 | CXCL11 |
| STAT1 | GBP1 |
| STAT1 | CXCL9 |
